# From bead to rod: Comparison of theories by measuring translational drag coefficients of micron-sized magnetic bead-chains in Stokes flow

**DOI:** 10.1371/journal.pone.0188015

**Published:** 2017-11-16

**Authors:** Kaiyuan Yang, Chen Lu, Xiaodan Zhao, Ryo Kawamura

**Affiliations:** 1 Mechanobiology Institute, National University of Singapore, 117411, Singapore, Singapore; 2 Center for Bio-Imaging Sciences, Department of Biological Sciences, National University of Singapore, 117543, Singapore, Singapore; 3 Department of Physics, National University of Singapore, 117542, Singapore, Singapore; The Ohio State University, UNITED STATES

## Abstract

Frictional drag force on an object in Stokes flow follows a linear relationship with the velocity of translation and a translational drag coefficient. This drag coefficient is related to the size, shape, and orientation of the object. For rod-like objects, analytical solutions of the drag coefficients have been proposed based on three rough approximations of the rod geometry, namely the bead model, ellipsoid model, and cylinder model. These theories all agree that translational drag coefficients of rod-like objects are functions of the rod length and aspect ratio, but differ among one another on the correction factor terms in the equations. By tracking the displacement of the particles through stationary fluids of calibrated viscosity in magnetic tweezers setup, we experimentally measured the drag coefficients of micron-sized beads and their bead-chain formations with chain length of 2 to 27. We verified our methodology with analytical solutions of dimers of two touching beads, and compared our measured drag coefficient values of rod-like objects with theoretical calculations. Our comparison reveals several analytical solutions that used more appropriate approximation and derived formulae that agree with our measurement better.

## Introduction

Fluid mechanics of particles in low Reynolds number flow, also referred to as creeping flow or Stokes flow [1, 2], plays central roles in the studies of microorganism locomotion such as cilia and flagella propulsion [3, 4], transportation of polymers in solution [5], biochemical macromolecules like DNA [6, 7] and cytoskeleton proteins [8], drug delivery [9], magnetic particles manipulation in micro-fluidic devices [10, 11], volcano ash settling in air [2], molecular dynamics simulation [12], fungal spores flight [13], and various chemical engineering applications [14]. As the name implies, the Reynolds number (Re) in these flows is very small (Re ≪ 1). Reynolds number is a dimensionless criterion useful for predicting the importance of inertial and viscous effects of the fluid [14]. It is defined to be the ratio of the inertial and viscous forces of the fluid, Re = *ρ***U***a*/*η*, where *ρ* is the density of the fluid, **U** is the relative velocity of the object to the flow, *a* is the characteristic length scale, and *η* is the viscosity of the fluid. Inertia is insignificant in Stokes or creeping flow, and viscous frictional effects dominate.

One salient feature of low Reynolds number flow is the simplification of the important but often complex Navier-Stokes equations. Solutions to the Navier-Stokes equation are generally hard to obtain, so approximations are often used for practical engineering analyses. In creeping flow (Re ≪ 1), approximate Navier-Stokes equation is reduced to [2]:

$$\begin{array}{c} \nabla P≅\eta \nabla^{2}U\;, \end{array}$$ (1)

with *P* being the fluid mechanical pressure. In this Stokes flow approximation, density is omitted in the Navier-Stokes equation, and the fluidic drag on an object becomes a function of its translational velocity **U**, characteristic length scale of the object *a*, and fluid viscosity *η* (for more discussion on Eq 2 please see Note A in S1 Appendix from the supplement):

$$\begin{array}{c} F^{drag}=-\text{constant}\cdot \eta Ua\;. \end{array}$$ (2)

Analytical exact solution exists for spherical objects with the characteristic length being its diameter 2*r*, and the constant term from Eq 2 turns out to be 3*π*. Thus the drag force on a sphere is given by

$$\begin{array}{c} F_{sphere}^{drag}=-3\pi \cdot \eta U2r=-6\pi \eta rU\;, \end{array}$$ (3)

which is commonly known as Stokes’ law [15]. More generally, the drag force on any 3-dimensional object undergoing translational motion in Stokes flow can be written as:

$$F^{drag}=-\xi \cdot U\;,$$ (4)

with *ξ* being the translational drag coefficient, which is proportional to the fluid viscosity and related to only the shape, size and orientation of the object [16]. As shown in Eq 3, the translational drag coefficient for spherical object in Stokes flow is therefore *ξ*_*sphere*_ = 6*πηr*. Drag coefficients for other geometric shapes have been analytically calculated in the past, such as for ellipsoid [17–21], cylindrical rod [18, 20, 22–25], two spheres [14, 26, 27], and chains of beads [5, 28]. For rod-like objects, the calculation has been especially tricky since there is extra consideration for end-effects and choice of geometrical approximation of the rod-like object. One popular model for rods with large aspect ratios is the rough approximation in the shape of a long thin ellipsoid or cylinder, and is often referred to as the “slender-body” theory [20, 22, 23]. Chain of beads modeling is also important in bead model theories of Kirkwood and Riseman [29], upon which theoretical works from García de la Torre *et al.* [25, 27, 30], Yamakawa and Tanaka [28], Doi and Edwards [5], and Swanson *et al.* [26] were developed. These theoretical representations of rod-like objects agree that drag coefficients of a rod is a function of its length and aspect ratio, but differ quite significantly among each other in the correction factors.

More recently, García de la Torre *et al.* developed a public-domain computer program, HYDRO++ [31, 32], based on the bead model theory. This simple software has been used to predict and calculate diffusion coefficients and other solution properties of nano-to-micron size particles [33, 34]. The bead model hydrodynamics implemented in HYDRO++ is also compared as one of the bead model theories to describe the properties of the present authors’ chain-of-bead microparticles.

A number of attempts have been made to quantify the drag coefficients of rod-like objects experimentally in the past, usually done by dropping millimeter-scale objects in a vessel [14, 35]. One problem with the traditional settling rates measurement is that the objects are too big for the vessels used to have no surrounding boundary and wall effects, which is problematic for interpretation of the experimental results. Orientation and accurate tracking of the objects are also difficult to achieve. As for experiments using more modern technology offering more precision and credibility, to the best of our knowledge, there are two. One is a short communication by Zahn *et al.* [36] that measured sedimentation speed of micron-scale bead-chains, but how they were able to use video-microscopy to observe and calculate the sedimentation speed is unclear. The other recent paper from Wise *et al.* [37] used similar methodology as ours, but their measurement, to quote their own words, contained several “spurious results”.

In our study, we measured the drag coefficients using magnetic tweezers, a technology that combines precise movement control of magnetic particles with accurate particle trajectory tracking [38]. Stokes’ law (Eq 3) has been used to calibrate magnetic force on magnetic particles since some of the first magnetic tweezers experiments [39, 40]. Magnetic beads used in this study are superparamagnetic and have low standard deviations in both geometric size and magnetic content [10, 41, 42]. The Reynolds number is also well within the range of Stokes flow in this case (*Re* < 10^−5^).

**Fig 1:**
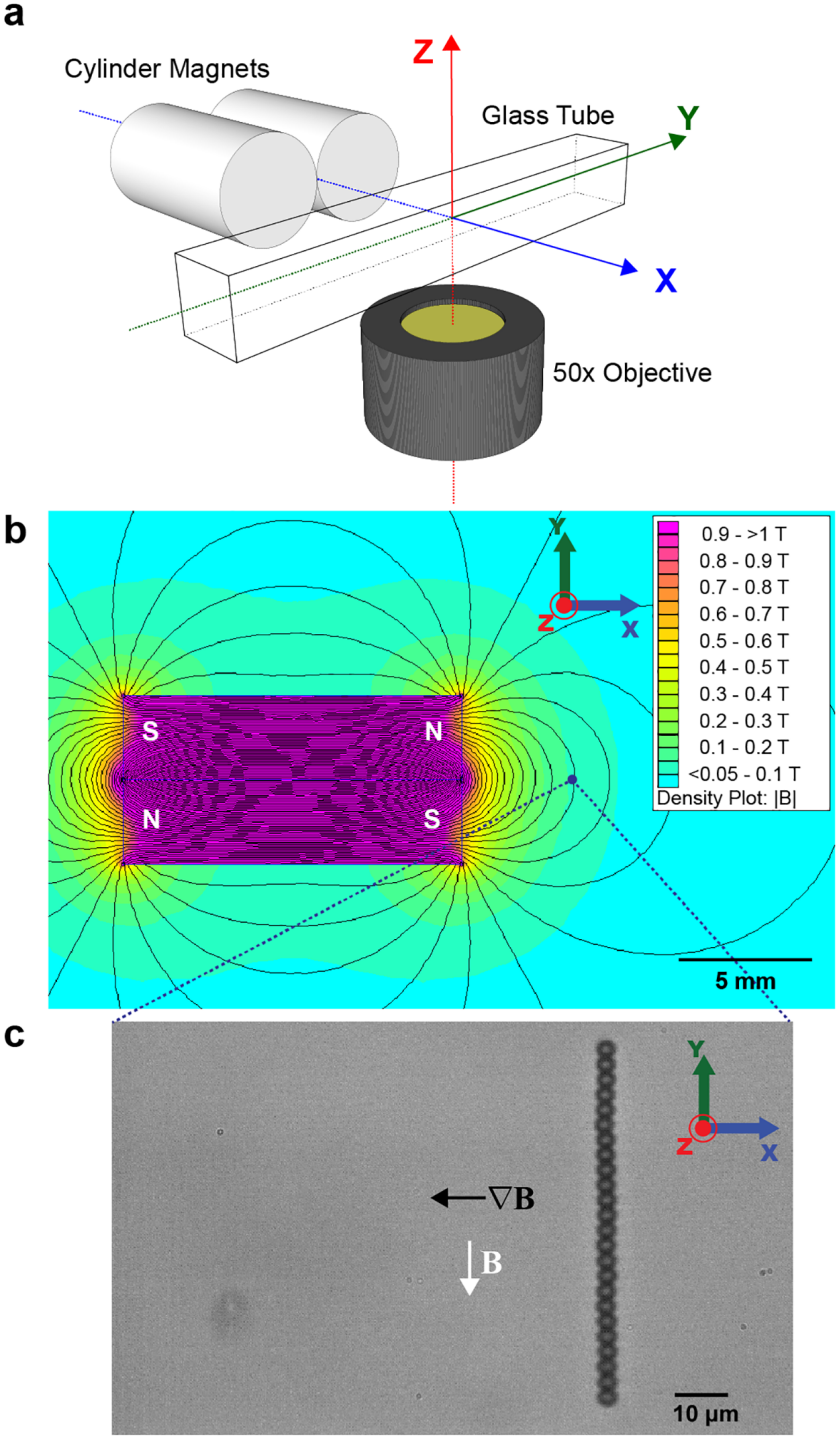
Magnetic tweezers setup for perpendicular translation of bead chains. (a): Schematic representation of the magnetic tweezers with a pair of cylinder magnets. The cylindrical magnets are magnetized in the axial direction, and they are paired up in an anti-parallel fashion. (b): Finite element simulation of the cylinder magnets pair using Finite Element Method Magnetics (FEMM 4.2). Cross section of the *x*, *y*-plane through the geometric center along *z*-axis is shown. Field density |**B**| decreases from pink to blue. N and S denote north and south poles respectively. Black lines represent computed magnetic field lines. Blue dot is a schematic field of view of the 50X objective, and blue dotted lines show zoom direction. Scale bar, 5 mm. (c): As viewed in the field of view, a 24-bead chain in the magnetic field of a pair of magnets, aligning with the field **B** (white arrow) along the *y*-axis, and translating towards the magnetic field gradient ∇**B** (black arrow). Scale bar, 10 μm.

**Fig 2:**
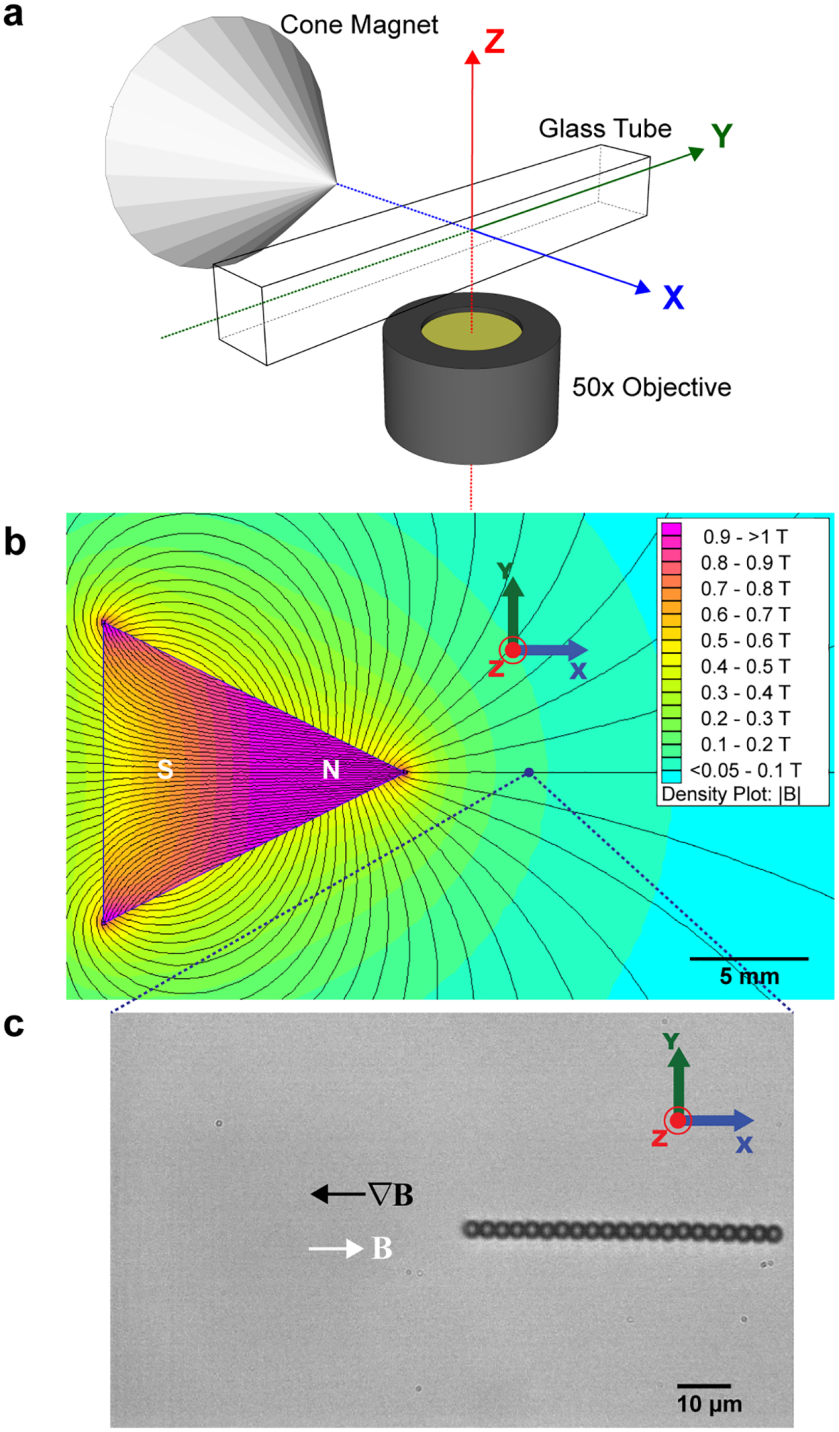
Magnetic tweezers setup for parallel translation of bead chains. (a): Schematic representation of the magnetic tweezers with a single cone magnet, which is magnetized north at tip and south at base. (b): Finite element simulation of the cone magnet using Finite Element Method Magnetics (FEMM 4.2). Cross section of the *x*, *y*-plane through the geometric center along *z*-axis is shown. Field density |**B**| decreases from pink to blue. N and S denote north and south poles respectively. Black lines represent computed magnetic field lines. Blue dot is a schematic field of view of the 50X objective, and blue dotted lines show zoom direction. Scale bar, 5 mm. (c): A 21-bead chain in the magnetic field of a single cone magnet, aligning with the field **B** (white arrow) along the *x*-axis, and translating towards the magnetic field gradient ∇**B** (black arrow). The force applied to the particle is in the direction of the magnetic field gradient ∇**B**. Scale bar, 10 μm.

We used a pair of cylindrical magnets, which are magnetized in the axial direction, to induce translational motion of bead-chains in the sidewise (perpendicular) orientation (Fig 1a). And we had a cone-shaped magnet, also magnetized in the axial direction, to generate translational motion of bead-chains in the lengthwise (parallel) orientation (Fig 2a). Here we report the measurement of translational drag coefficients in Stokes flow regime of bead-chains moving either parallel or perpendicular to the axial axis. We also compare our experimental values with theories describing hydrodynamic properties of rod-like objects, namely bead model, ellipsoid model, and cylinder model.

## 1 Materials and methods

### Glycerol and magnetic beads suspension

Superparamagnetic beads with diameter of 2.8 μm were obtained from Dynabeads M270, Invitrogen, USA. Analytical reagent grade glycerol 99+% was from Fisher Scientific, UK. Glycerol was either used as 99% stock or 50% dilution with DI water for mixing with M270 magnetic beads. For a few hours before experiment, glycerol solution was incubated on benchtop to allow large trapped air-bubbles to escape, and magnetic beads were introduced to glycerol and mixed gently to prevent air-bubbles formation. Suspension had final concentration of 600 to 700 /μl magnetic beads, equivalent to 6-7 beads per 10^7^ μm^3^ of suspension volume. Beads concentration was kept low to minimize flow pattern and magnetic interactions from nearby particles to the particle being tracked in the camera view. In the glass tube, only particles moving in the central region (at least 100 μm distance from the upper and lower walls) were selected to be tracked in the focal plane such that the drag coefficient was not influenced by nearby walls.

### Magnetic tweezers setup

An in-house made transverse magnetic tweezers setup was used in this study [43–45]. A miniature borosilicate glass tube (VitroTubes 8250, VitroCom, USA) with inside chamber dimensions of 0.5 mm, 0.5 mm and 25 mm and wall thickness of 0.1 mm was placed on an inverted microscope (Olympus CKX41) (Figs 1a and 2a). The inner chamber of the glass tube was filled with glycerol suspended with trace amount of magnetic particles. There was no fluid flow in the inner chamber of the VitroTube, as the setup was intended for stationary fluids except for the displacement of magnetic particles.

Magnetic forces were applied to superparamagnetic particles (beads and their chain formations) via either a pair of cylinder-shaped permanent magnets (Neodymium material grade N52, 3 mm diameter and 12.7 mm height, KJ Magnetics, USA) (Fig 1a) or a single cone-shaped permanent magnet (Neodymium material grade N50, 12.7 mm diameter and 12.7 mm height, SuperMagnetMan, USA) (Fig 2a). In our design as sketched in Figs 1 and 2, since the geometric center of magnets, either a pair of cylinder or a single cone magnet, was aligned with the center of the camera view, this direction is defined as the *x*-direction. The lightpath to the objective and CCD camera is defined as the *z*-direction. Then, the *y*-direction, which is perpendicular to the *xz* plane, is defined in the Cartesian coordinate.

Magnetic field density plot along the *x*, *y*-plane at the geometric center of magnets in *z*-axis is shown for both setups (Figs 1b and 2b). The magnetic field simulation was done using an open-source software, Finite Element Method Magnetics (FEMM 4.2), in which computed heatmap of the external magnetic field **B** from the magnet(s) and approximated magnetic field lines were plotted (see Theory/Calculations).

While the pair of cylinder magnets induced the formation of bead-chains orientated perpendicular to the *x*-axis (Fig 1c), cone magnet generated chains orientating parallel along the *x*-axis (Fig 2c). Magnets were mounted on a step motor controlled by MP285 manipulator (Sutter Instrument, USA), which can move in 3-dimensions with a 40 nm stepping accuracy. We tuned the *z* position of the magnets so the magnetic force generated was parallel to the plane of focus, and the motion of the particles was along the focal plane.

In the external magnetic field, magnetic moment of a single bead generates a stray magnetic induction that attracts other nearby magnetic beads to chain up along the magnetic field line. In our experimental setting, the beads concentration was kept low to minimize influence from magnetic induction field of other magnetic beads and their formed chains. Therefore in our experiments, we initially observed single beads. And with external magnetic field and gradual diffusion process coupled with induced movement towards magnets, magnetized beads moved in range of stray induction field of other beads and form linear chains. So with time, higher percentage of beads would form chain structures and with longer chain length. Nonetheless these beads are superparamagnetic, and they lose their magnetization when no external magnetic field is present (no remanence and hysteresis). With removal of magnets, the magnetic chain structures disjointed into individual beads.

### Magnetic particle tracking

**Fig 3:**
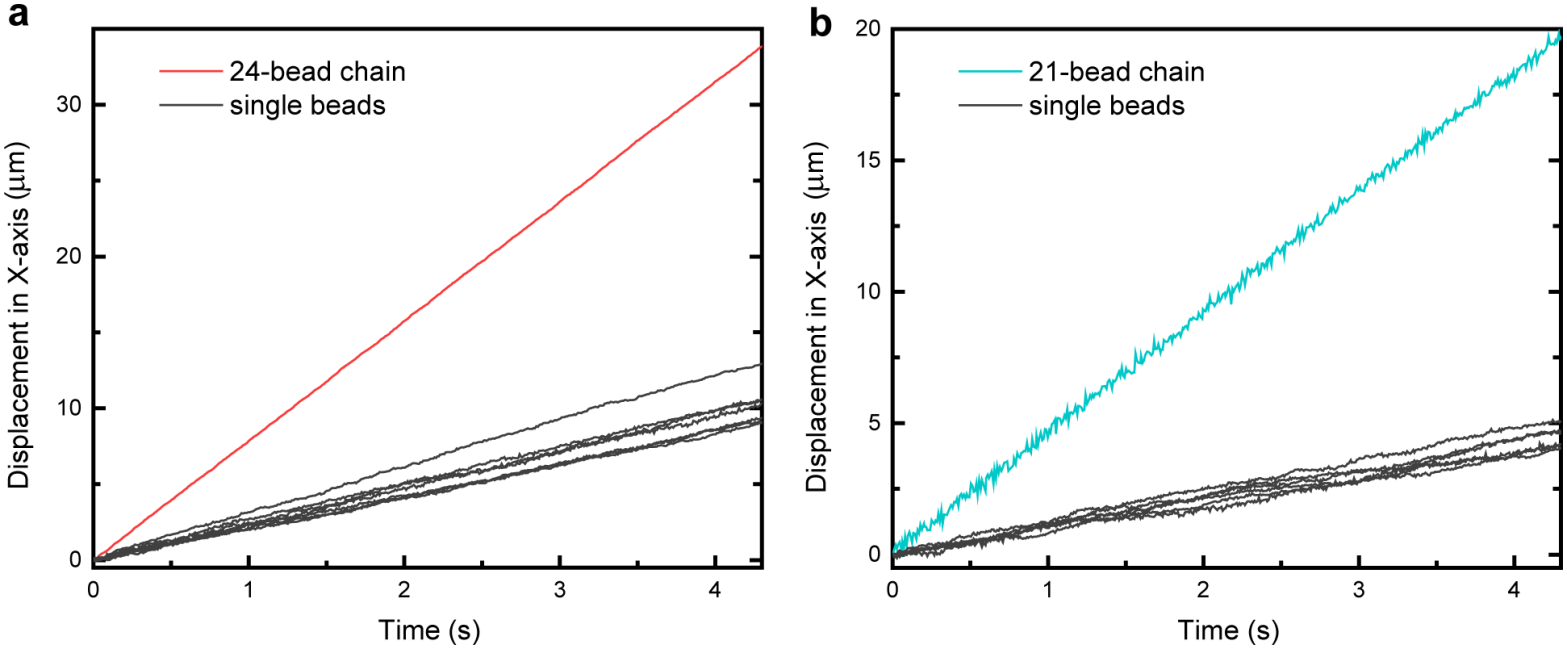
Magnetic beads and bead-chains tracking with LabVIEW program. (a): Displacement along the *x*-axis of the 24-bead chain from Fig 1c versus time plotted in red. Several single beads measured in the same experiment plotted in black. (b): Displacement along the *x*-axis of the 21-bead chain from Fig 2c versus time plotted in cyan. Several single beads measured in that same experiment plotted in black. The velocity of a given bead or bead-chain was calculated by dividing the displacement along *x*-axis by the time taken.

Magnetic particles in the glass tube were bright-field-imaged with 50X magnification and tracked at a sampling rate of 100Hz (Figs 1c and 2c). Their positions were determined by centroids with a resolution of 30 nm [46], using a home-written LabVIEW program (National Instruments, USA). More information on the LabVIEW image processing codes can be found in Fig A in S1 Appendix. For the same fixed magnet position (1000 μm to 12 000 μm) in any one experiment setting, trajectories of individual beads or bead-chains along *x*-axis were plotted against time as shown in Fig 3. Velocity of a given magnetic particle was calculated as **U** = *d***x**/*dt*, where **x** is the displacement along *x*-axis and *t* is time recorded. Single beads’ velocities in a particular experiment were collected as reference and used to calculate the drag coefficients of bead-chains in that same experiment. In total, eight experiments were conducted to obtain a range of bead-chains varying in chain length from 2 to 27. All experiments were conducted at 25 ± 1°C. Short videos of these translational motions of magnetic particles described in the Method can be found in S1–S3 Videos in supporting information.

## 2 Theory/Calculations

### Magnetic force and drag force

Magnetic particles are influenced by the presence of an external magnetic field **B** both in terms of torque and magnetic force. The field will rotate and align the particle’s magnetic moment **M** with the field line with a torque ***τ*** = **M** × **B**. However, a uniform field only generates torque and a magnetic field gradient ∇**B** is needed to exert magnetic force and induce translational motion on the suspended particle. For the superparamagnetic M270 Dynabead used in this study, the magnetic force on a single bead with magnetic moment **m** is quantified as:

$$F_{1}^{mag}=\left(m\cdot \nabla \right)B\;.$$ (5)

**Fig 4:**
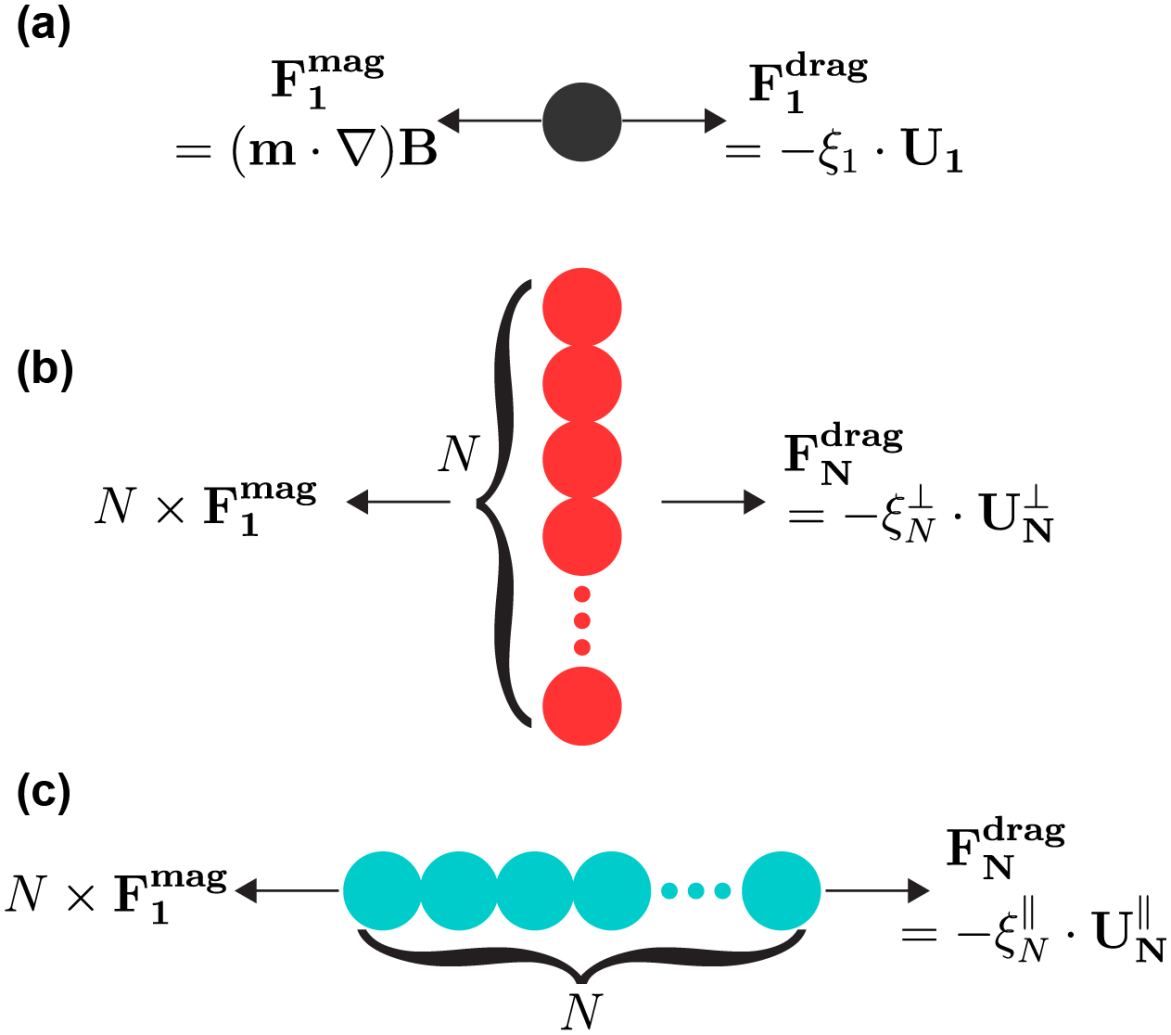
Principle of drag coefficient calculations. Schematic illustration of magnetic force and drag force acting on (a) single bead, (b) bead-chain moving perpendicular to its axial axis, and (c) bead-chain moving parallel to its axial axis. For a given bead-chain of length *N*, the drag coefficient is calculated using Eqs 9 and 11.

This results in translation towards regions of higher magnetic field along *x*-axis. And opposite the direction of translation is the frictional drag force:

$$\begin{array}{c} F_{1}^{drag}=-\xi_{1}\cdot U_{1}\;, \end{array}$$ (6)

where **U**_1_ is the translational velocity of the spherical bead with radius *r* and *ξ*_1_ = 6*πηr* is the single bead’s drag coefficient (Fig 4a).

One thing to note is the influence of a solid wall in the vicinity of the moving particle. The drag coefficient of a spherical bead near a wall surface is approximated by Faxén’s law [47]:

$$\begin{array}{c} \xi_{1}=\frac{\xi_{sphere}}{1-\frac{9r}{16h}+\frac{r^{3}}{8h^{3}}-\frac{45r^{4}}{256h^{4}}-\frac{r^{5}}{16h^{5}}}\;, \end{array}$$ (7)

where *ξ*_*sphere*_ = 6*πηr* is the theoretical drag coefficient when the bead is far away from the tubing wall, *h* is the distance of the geometric center of the bead from wall, and *r* is the radius of the bead. For bead right on surface, *ξ*_1_ ≈ 3.1 × *ξ*_*sphere*_. For bead much far away from wall surfaces (i.e. *h* ≫ *r*), *ξ*_1_ ≈ *ξ*_*sphere*_.

The magnetic bead-chain formed in the magnetization of the pair of cylinder magnets (Fig 1) is schematically shown in Fig 4b. The translational motion is perpendicular to the axial axis of the chain, and we shall denote this translation orientation of the chain as ⊥. For a given magnetic particle, the magnetic force applied to the particle only depends on the distance between the particle and the magnets placed outside the glass tube. Given that in our experiments, the magnets were typically positioned more than 1000 μm from the objective view, magnetic force applied to particles can be assumed to be effectively constant over the range (travel distance of <40 μm) of translation recorded for the measurement (see Results and discussion for the error analysis). For the same magnet position that gives rise to $F_{1}^{mag}$ on a single bead, with *N* beads in a chain, the magnetic force on the chain is balanced by the drag force:

$$\begin{array}{c} N\times F_{1}^{mag}=-\xi_{N}^{⊥}\cdot U_{N}^{⊥}\;, \end{array}$$ (8)

and since $F_{1}^{mag}=F_{1}^{drag}$, the perpendicular translational drag coefficient can be written as:

$$\begin{array}{c} \frac{\xi_{N}^{⊥}}{\xi_{1}}=\frac{N\cdot U_{1}}{U_{N}^{⊥}}\;. \end{array}$$ (9)

For the magnetization by the cone-shaped magnet (Fig 2), bead-chain formation is depicted in Fig 4c. Translational motion is parallel to the axial axis of the chain, and this translation orientation of the chain is marked as ∥. And similarly, for a chain with *N* beads, magnetic force equals to the drag force:

$$\begin{array}{c} N\times F_{1}^{mag}=-\xi_{N}^{∥}\cdot U_{N}^{∥}\;, \end{array}$$ (10)

and the parallel translational drag coefficient is given by:

$$\begin{array}{c} \frac{\xi_{N}^{∥}}{\xi_{1}}=\frac{N\cdot U_{1}}{U_{N}^{∥}}\;. \end{array}$$ (11)

For a given bead-chain of length *N*, the drag coefficient can be measured by determining the velocity of the chain **U**_*N*_ and the average of velocities of single beads 〈**U**_1_〉 in the same experiment (Fig 3).

### Theoretical drag coefficients for rod-like objects

Theoretical equations describing the drag coefficients in parallel or perpendicular motion to the axial axis of the rod were generally based on three geometrical representations of the rod-like object: bead model, ellipsoid model, and cylinder model.

Bead model is a rigid array of *N* touching spherical beads, each with radius *r*. Yamakawa and Tanaka [28] and Doi and Edwards [5] derived drag coefficients of rigid rod-like polymers using such bead model.

In ellipsoid model, the rod-like object is approximated to be a slender prolate ellipsoid with major axis *L*/2 and minor axis *r*. Oberbeck [17] and Burgers [18, 20] first calculated the flow around such ellipsoid, and their analyses were revisited by Tchen [19], Cox [20] and Chwang and Wu [21] among others.

For cylinder model, the rod is modeled as a cylinder with length *L* and cross-sectional diameter of 2*r*. Calculations for drag coefficients based on such a rigid long cylinder rod were first proposed by Burgers [18, 23], followed by theoretical development from Cox [20], Batchelor [23], Tillett [22], Broersma [24], and Tirado *et al.* [25].

All three models have derived the same basic form of equation relating drag force to a function of the length *L* of the rod and its aspect ratio *L*/(2*r*):

$$\begin{array}{c} F_{∥}^{drag}=-\xi_{∥}U_{∥}=-\frac{2\pi \eta L}{\ln\left(\frac{L}{2r}\right)+\gamma_{∥}}U_{∥}\;, \end{array}$$ (12)

$$\begin{array}{c} F_{⊥}^{drag}=-\xi_{⊥}U_{⊥}=-\frac{4\pi \eta L}{\ln\left(\frac{L}{2r}\right)+\gamma_{⊥}}U_{⊥}\;, \end{array}$$ (13)

where the only term that differs among the various theoretical calculations is the correction factor *γ*_∥_ and *γ*_⊥_. The values of *γ*_∥_ and *γ*_⊥_ are summarized in Table 1.

**Table 1:** Correction factor *γ*_∥_ and *γ*_⊥_ from various theories for rod-like object.

Model	Author	*γ*_∥_	*γ*_⊥_
Bead	Yamakawa [28]	0.044	1.111
	Doi & Edwards [5]	0	0
Ellipsoid	Oberbeck [17]	0.193	1.193
	Burgers [18]		
	Tchen [19]		
	Cox [20]		
	Chang & Wu [21]		
Cylinder	Burgers [18]	−0.027	1.193
	Cox [20]	−0.114	0.886
	Tillett [22]	−0.114	0.886
	Batchelor [23]	−0.114^a^	0.886^a^
	Broersma [24]	Eq 18	Eq 19
	Tirado [25]^b^	Eq 16	Eq 17

^a^ Batchelor [23] obtained identical correction factors *γ* as Tillett [22] and Cox [20], and he also provided more exact forms of the equations in Eqs 20 and 21.

^b^ The theory in Tirado [25] has been later updated to cover even shorter cylinders and disks [48].

Clearly, all three geometrical representations of the rod-like object are related to *N* both as the number of beads in a bead-chain and the aspect (or length-to-thickness) ratio *L*/(2*r*). If we normalize the drag coefficient in Eqs 12 and 13 to that of a single bead with diameter 2*r*, *ξ*_1_ = 6*πηr*, we obtain:

$$\begin{array}{c} \frac{\xi_{∥}}{\xi_{1}}=\frac{2}{3}\frac{N}{\ln\left(N\right)+\gamma_{∥}}\;, \end{array}$$ (14)

$$\begin{array}{c} \frac{\xi_{⊥}}{\xi_{1}}=\frac{4}{3}\frac{N}{\ln\left(N\right)+\gamma_{⊥}}\;. \end{array}$$ (15)

So effectively we have rewritten the drag coefficient of a rod-like object as a function of *N* alone.

In fact, correction factors *γ*_∥_ and *γ*_⊥_ proposed by Tirado [25] and Broersma [24] also come as functions of *N* as well. Tirado *et al.* [25] predicted that

$$\begin{array}{c} \gamma_{∥}=-0.207+0.980/N-0.133/N^{2}\;, \end{array}$$ (16)

$$\begin{array}{c} \gamma_{⊥}=0.839+0.185/N+0.233/N^{2}\;, \end{array}$$ (17)

and Broersma’s equations give

$$\begin{array}{c} \begin{array}{cc} \gamma_{∥}= & -0.114-0.15/\ln\left(2N\right)-13.5/{\left(\ln\left(2N\right)\right)}^{2}\\ & +37/{\left(\ln\left(2N\right)\right)}^{3}-22/{\left(\ln\left(2N\right)\right)}^{4}\;, \end{array} \end{array}$$ (18)

$$\begin{array}{c} \begin{array}{cc} \gamma_{⊥}= & 0.866-0.15/\ln\left(2N\right)-8.1/{\left(\ln\left(2N\right)\right)}^{2}\\ & +18/{\left(\ln\left(2N\right)\right)}^{3}-9/{\left(\ln\left(2N\right)\right)}^{4}\;. \end{array} \end{array}$$ (19)

Batchelor also provided the drag coefficient of a cylindrical rod in more exact forms:

$$\begin{array}{c} \frac{\xi_{∥}}{\xi_{1}}=\frac{2}{3}N(\frac{\epsilon +0.307\epsilon^{2}}{1-\epsilon /2}+0.426\epsilon^{3})\;, \end{array}$$ (20)

$$\begin{array}{c} \frac{\xi_{⊥}}{\xi_{1}}=\frac{4}{3}N(\frac{\epsilon +0.307\epsilon^{2}}{1+\epsilon /2}+0.119\epsilon^{3})\;, \end{array}$$ (21)

where *ϵ* = 1/ln(2*N*).

## 3 Results and discussion

Theoretical solutions for the translational drag coefficients of two touching spheres of the same size in Stokes flow have been discussed extensively in literature such as Happel and Brenner [14], Carrasco [27], and Swanson *et al.* [26]. The dimer bead model described in Carrasco [27] has been further developed into a comprehensive bead modelling methodology [32], which is implemented in the public-domain computer program HYDRO++ [31, 32]. Such a dimer of identical spheres correspond to our measurements of 2-bead chains. The results for dimer from our magnetic tweezers experiments are listed alongside theoretical calculations in the Row 1 of Table 2. Good agreement is obtained for both parallel and perpendicular translational drag coefficients. This confirmation of our experimental results for the case of two touching spheres validates the accuracy of our experimental method.

**Table 2:** Comparison of experimental and theoretical values *ξ*_*N*_/*ξ*_1_ for bead-chains of length 2 to 30.

Chain Length N	Experimental^a^		Swanson [26]		Durlofsky [49]		HYDRO++^b^	
	$\xi_{N}^{∥}/\xi_{1}$	$\xi_{N}^{⊥}/\xi_{1}$	$\xi_{N}^{∥}/\xi_{1}$	$\xi_{N}^{⊥}/\xi_{1}$	$\xi_{N}^{∥}/\xi_{1}$	$\xi_{N}^{⊥}/\xi_{1}$	$\xi_{N}^{∥}/\xi_{1}$	$\xi_{N}^{⊥}/\xi_{1}$
2	1.28 ± 0.03 (20)	1.45 ± 0.03 (24)	1.29	1.43	…	…	1.23	1.39
3	1.54 ± 0.03 (16)	1.83 ± 0.04 (23)	…	…	…	…	1.49	1.78
4	1.76 ± 0.04 (14)	2.07 ± 0.04 (22)	…	…	…	…	1.71	2.13
5	2.00 ± 0.03 (16)	2.26 ± 0.06 (18)	…	…	1.99	2.16^c^	1.92	2.46
6	2.18 ± 0.05 (10)	2.64 ± 0.06 (21)	…	…	…	…	2.12	2.78
7	2.42 ± 0.08 (7)	3.13 ± 0.10 (16)	…	…	…	…	2.32	3.09
8	2.51 ± 0.06 (6)	3.37 ± 0.20 (5)	…	…	…	…	2.50	3.39
9	2.79 ± 0.04 (10)	3.48 ± 0.12 (7)	…	…	…	…	2.69	3.68
10	2.80 ± 0.09 (8)	3.74 ± 0.15 (6)	…	…	2.96	3.66	2.86	3.96
11	2.97 ± 0.08 (4)	4.08 ± 0.26 (3)	…	…	…	…	3.04	4.25
12	3.09 ± 0.19 (3)	4.08 (1)	…	…	…	…	3.21	4.52
13	3.17 ± 0.14 (5)	4.81 ± 0.09 (6)	…	…	…	…	3.38	4.79
14	3.14 ± 0.12 (9)	4.86 (1)	…	…	…	…	3.54	5.06
15	3.52 ± 0.11 (2)	5.04 ± 0.19 (2)	…	…	3.83	5.06	3.70	5.32
16	…	5.61 (1)	…	…	…	…	3.86	5.58
17	3.84 (1)	…	…	…	…	…	4.02	5.84
18	4.52 ± 0.02 (2)	…	…	…	…	…	4.18	6.10
19	4.03 (1)	6.37 ± 0.04 (2)	…	…	…	…	4.33	6.35
20	…	…	…	…	4.64	6.36	4.48	6.60
21	4.53 (1)	…	…	…	…	…	4.63	6.85
22	…	…	…	…	…	…	4.78	7.09
23	…	…	…	…	…	…	4.93	7.34
24	4.43 (1)	7.77 (1)	…	…	…	…	5.08	7.58
25	3.86 (1)	…	…	…	5.43	7.63	5.23	7.82
26	5.08 (1)	…	…	…	…	…	5.37	8.06
27	…	8.51 (1)	…	…	…	…	5.51	8.29
28	…	…	…	…	…	…	5.66	8.53
29	…	…	…	…	…	…	5.80	8.76
30	…	…	…	…	6.15	8.85	5.94	9.00

^a^ Mean and variance of ratio obtained from *ξ*_*N*_ and *ξ*_1_ and calculated with Taylor series expansion [50, 51]. Data are shown in mean ± s.e.m. (number of measured bead-chains).

^b^ Public-domain computer program HYDRO++ [31, 32]. See Note B in S1 Appendix for more details on the software usage.

^c^ This $\xi_{N}^{⊥}/\xi_{1}$ column is obtained after end-removal or “*N* − 2” treatment.

In Row 2 onwards of Table 2, we present the experimental results for bead-chain of length 2 to 27, compared with theoretical calculations of $\xi_{N}^{∥}/\xi_{1}$ and $\xi_{N}^{⊥}/\xi_{1}$. Chain lengths of short (*N* < 5) range are often excluded for application of equations on rod-like objects [20, 23–25, 28]. More importantly, no analytical solutions are available for such short arrays of beads [27] or for short cylinders [14].

Computational solutions, however, have been proposed for short chain length in this range (and beyond). Durlofsky *et al.* [49] computed using simulation the drag on linear chains of *N* touching spheres and the shortest chain length attempted was five, as shown in Row 4 of Table 2. Comparison of parallel drag coefficient *ξ*_∥_/*ξ*_1_ yields reasonable agreement. Durlofsky *et al.* provided two sets of computation results, one with drag from the two beads at either extremity removed, and one without such treatment. Interestingly, for parallel translational drag coefficient, without removal of drag contribution from two ends gives better conformity to our measurement. Perpendicular drag coefficient *ξ*_⊥_/*ξ*_1_, however, seems to require two-end removal to improve the match to our measurement data. This is in relation to the larger correction factor *γ*_⊥_ needed for perpendicular translations than parallel ones *γ*_∥_ as shown in Table 1. In fact, Tirado *et al.* [25] and Broersma [24] referred to *γ* as end-effect corrections. This usage of the term combined with our experiment with computation from Durlofsky *et al.* [49] suggest that end of the rod contribute more to drag coefficient in perpendicular translation than parallel motion, and resulting in larger end-effect correction in the form of *γ*. The computational solutions provided by HYDRO++ program not only predict quite accurately the translational drag coefficients for short chain length, but are also able to describe the drag coefficients of moderately long bead-chains as seen in Table 2.

**Fig 5:**
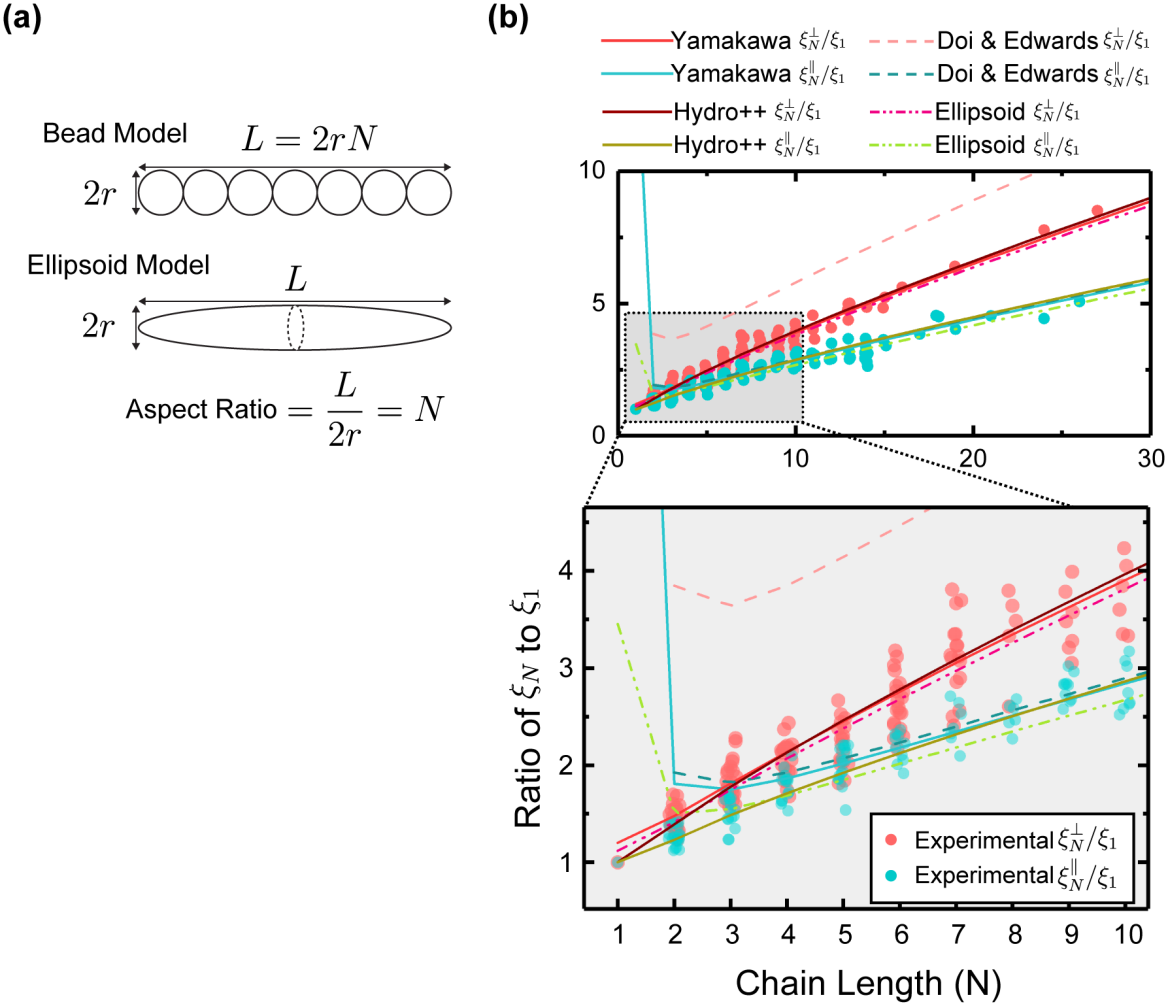
Bead model and ellipsoid model theories and measurements. (a): Schematic of the bead model and ellipsoid model theoretical frameworks representing rod-like objects. Bead model is a rigid array of identical touching beads each with diameter 2*r*; ellipsoid model is composed of major axis *L*/2 and minor axis *r*; Chain length in bead model in terms of number of beads *N* is equivalent to aspect ratio of ellipsoid *L*/(2*r*). (b): Experimental values for $\xi_{N}^{⊥}/\xi_{1}$ (red color dots) plotted along with experimental values for $\xi_{N}^{∥}/\xi_{1}$ (cyan color dots) versus chain length *N*. Theoretical curves are from Eqs 14 and 15 substituted with appropriate values of *γ* based on bead model and ellipsoid model as listed in Table 1, and HYDRO++ values in Table 2.

**Fig 6:**
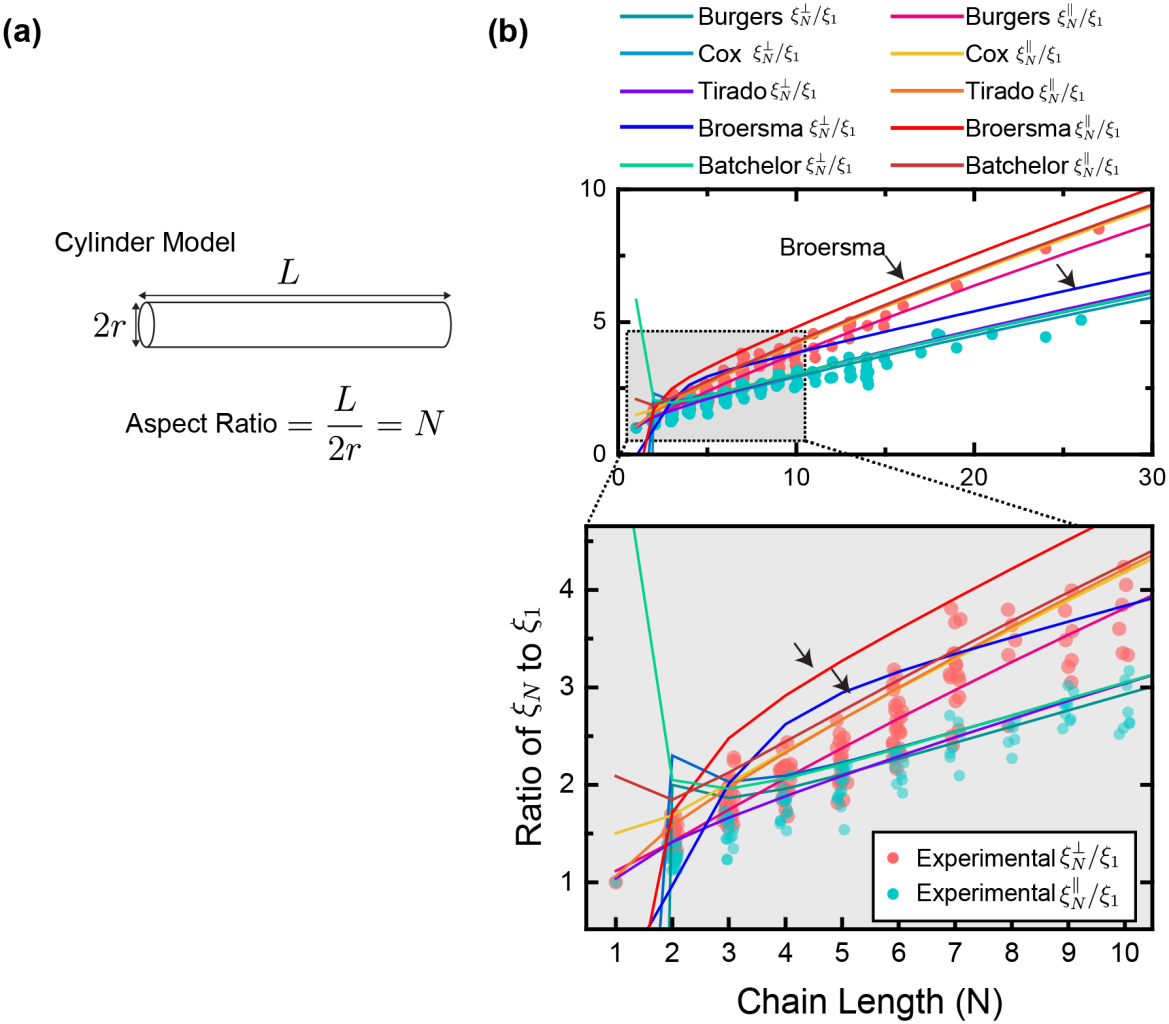
Cylinder model theories and measurements. (a): Schematic of the cylinder model theoretical framework representing rod-like objects. Cylinder model has diameter of 2*r* and length of *L*. Chain length in bead model in terms of number of beads *N* is equivalent to aspect ratio of ellipsoid and cylinder *L*/(2*r*). (b): The same experimental data from Fig 5b compared with theoretical curves with various *γ* from cylinder model as listed in Table 1. Black arrows point to theoretical curves obtained from Broersma [24].

In Figs 5 and 6 we compare experimental data of *ξ*_*N*_/*ξ*_1_ obtained in our study with the theoretical equations Eqs 14 and 15 substituting appropriate correction factors *γ*_∥_ and *γ*_⊥_ listed in Table 1, and HYDRO++ results from Table 2. Theoretical curves for bead model and ellipsoid model were plotted in Fig 5, and various plots of equations for cylindrical rod model were shown in Fig 6.

The dispersion of experimental points in Figs 5 and 6 could be due to several reasons. One is the slight heterogeneity of magnetic beads, especially their magnetic response [41, 52]. The magnetization of these beads used in the present study arises from iron oxide nanoparticles (10 − 20nm) embedded inside the plastic polymer, and these tiny magnetic units are distributed randomly in and among the beads [42]. Our group previously measured the heterogeneity in magnetization to be around 10% [52] and other magnetic tweezers group has reported the variation between beads to be 7% [41]. During single bead calibration for *ξ*_1_, we noted the magnetic response from the beads at the same magnet position to vary by 10% as well. And this variation is related to another source of error in our experiment, which is the variation of magnetic force over the field of view (FOV). The size of our FOV is 126 μm x 77 μm. For the velocity tracking of the particles, although we rarely picked particles quite far away from the center of the view (assuming furthest distance apart to be 60 μm), and these particles never traveled a prolonged distance across the FOV (smaller than 40 μm), we estimated an uncertainty of about 6% or less into the magnetic force value, by substituting *d* = 60 μm based on the magnetic force formula in [52]. Differences in both the magnetization of the beads and their magnetic force due to being tracked in different regions of FOV was reflected in the variation in single bead velocities in Fig 3. The experimental measurements in this study were conducted with multiple repeats by finding more bead-chains of certain length if possible, as shown in Table 2. And the means obtained from more repeated experiments generally showed meaningful agreement with certain theoretical predictions while deviated more from others.

All three bead models, HYDRO++ program [31, 32], Yamakawa [28], and Doi & Edwards [5] provided solutions for the flow around rod-like object represented as bead chains (Fig 5). However, Doi and Edwards [5] suggested their equations without any correction factors *γ*. As we have observed from our comparison with Durlofsky *et al.* [49]’s simulation above, drag effect from two ends of the rod-like object is more prominent in perpendicular translation than in parallel motion. End-effect correction is needed for *γ*_⊥_, which is shown in curve that has much higher $\xi_{N}^{⊥}/\xi_{1}$ than experimental values, given by Doi and Edwards [5]. On the contrary, Yamakawa [28] derived *γ*_∥_ and *γ*_⊥_ that closely fit with our measurement for *N* > 5. End-effect correction for parallel drag coefficient of the rod appears to be negligible, as shown in the satisfactory agreement between measured $\xi_{N}^{∥}/\xi_{1}$ and the ones proposed by Yamakawa [28] and Doi and Edwards [5], with *γ*_∥_ of 0.044 and 0 respectively. Both Yamakawa [28] and Doi [5] based their bead models on Kirkwood method [29], which itself requires approximation to get analytical results. Our comparison of theory with measurement reveals that Yamakawa [28] seems to use a better approximation and obtain a $\xi_{N}^{⊥}/\xi$ that agrees with experimental values better, while both Yamakawa and Doi suggested very good $\xi_{N}^{∥}/\xi$ solutions. HYDRO++ prorgam provides a better agreement with experiments over the whole range of chain length from short to moderately long rods.

Ellipsoid model also shows a very good match between its predicted values and experimental observations (Fig 5) over much of the experimental data range. *ξ*_*N*_/*ξ*_1_ prediction is fairly accurate for *N* bigger than five. Quantitatively, ellipsoid model derives correction factors *γ* very close to that by Yamakawa [28]’s bead model, and qualitatively, both provide very reasonable models of a rod-like object in Stokes flow for *N* generally larger than five.

Cylinder model tend to overestimate the perpendicular $\xi_{N}^{⊥}/\xi_{1}$ and, although slightly, parallel $\xi_{N}^{∥}/\xi_{1}$ translational drag coefficients of the bead-chains measured in our experiment, except for the $\xi_{N}^{⊥}/\xi_{1}$ calculated by Burgers [18] (Fig 6). Deviation of the theory and experiment is especially obvious for Broersma [24] with both $\xi_{N}^{⊥}/\xi_{1}$ and $\xi_{N}^{∥}/\xi_{1}$ curves above the measured values. Exact solutions to creeping motion equations for finite cylinders have been known to be difficult to obtain [14]. Swanson [26] calculated drag coefficients specifically for short cylinders and proposed that $\xi_{4}^{∥}/\xi_{1}=1.95$ and $\xi_{4}^{⊥}/\xi_{1}=2.4$, when our measured data are 1.76 and 2.07 respectively. Arguably the geometrical representations of a 4-bead chain versus a cylinder with an aspect ratio of 4 are different enough to produce drag coefficients this far apart. However, even for when the aspect ratio/chain length *N* is considerably large (*N* > 10), cylinder model still tend to predict higher drag coefficients than what we measured, more so for *ξ*_⊥_ (except Burgers [18]).

With the use of magnetic tweezers and CCD-camera based particle tracking we have conducted a careful measurement of the translational drag coefficients of chains of magnetic beads with length or aspect ratio of *N* from 2 to 27. These magnetic particles are of the size between 2.8 μm to smaller than 80 μm in a microtube’s inner chamber that is at least 6 × 10^8^ larger than these particles in volume, effectively creating an unbounded fluid condition crucial for theoretical calculations [14]. For dimers consisting of two beads, we validated past theoretical predictions by comparing with our measurement. And with such verification we presented drag coefficients for other short chains that are usually not considered for formulae on rod-like objects.

## Conclusion

Analytical and computational solutions for rod-like objects have been built around geometrical frameworks modeling these rods as an array of beads, an ellipsoid, or a cylinder. These theories are often rough approximations of rod-like objects that can be of arbitrary shape. Different theories provide different correction factors *γ* based on how geometrical conditions are treated in their models. The comparison between our experimental data and these theoretical frameworks have revealed several interesting characteristics. Perpendicular drag coefficient *ξ*_⊥_ of rod-like objects is best captured by bead model from Yamakawa [28], the ellipsoid model, and Burgers [18] of cylinder model. Parallel drag coefficient *ξ*_∥_ is best described by both Yamakawa and Doi’s bead models, the ellipsoid model, all cylinder models for *N* > 5 except Broersma [24]. And for both perpendicular and parallel coefficients, HYDRO++ program [31, 32], with its comprehensive and more advanced bead modeling scheme, is shown to describe the hydrodynamic translational drag coefficients with the best agreement over the entire rod aspect ratio measured.

We have presented translational drag coefficients in both the parallel and perpendicular components defined by the orientation of the rod axial axis to the direction of the flow. Hopefully we have helped to provide a more precise understanding of the components of the drag and related diffusion coefficients of rod-like objects that may assist in constructing a more accurate model for both theoretical and application works in Stokes flow.

## Supporting information

S1 Appendix Document containing Notes A-B and Fig A. (PDF)

S1 Video Video of single-bead movement. For more information on videos, please refer to S1 Appendix.
(AVI)

S2 Video Video of perpendicular translation of bead-chains. Shown in this video is a magnetic bead-chain of length *N* = 4 translating in the magnetic field by a pair of anti-parallel cylindrical magnets.
(AVI)

S3 Video Video of parallel translation of bead-chains. Parallel translation of two bead-chains of length *N* = 10 in the magnetic field due to a cone-shaped magnet.
(AVI)
