# Supplementary material for: From bead to rod: Comparison of theories by measuring translational drag coefficients of micron-sized magnetic bead-chains in Stokes flow
Source: PLoS One. 2017 Nov 16;12(11):e0188015. doi: 10.1371/journal.pone.0188015 (PMC5690466; doi:10.1371/journal.pone.0188015)
Supplement: S1 Appendix — (PDF) [file pone.0188015.s001.pdf]

# From bead to rod: Comparison of theories by measuring translational drag coefficients of micron-sized magnetic bead-chains in Stokes flow

Kaiyuan Yang[1,2*], Chen Lu[1,2], Xiaodan Zhao[1,2], Ryo Kawamura[2,3]

**1** Mechanobiology Institute, National University of Singapore, 117411, Singapore
**2** Center for Bio-Imaging Sciences, Department of Biological Sciences, National University of Singapore, 117543, Singapore
**3** Department of Physics, National University of Singapore, 117542, Singapore

* Corresponding author Email: mbiykai@nus.edu.sg

## Supporting Information

### Note A

For more comprehensive information on mathematical approximation of the constant term in Equation 2, please refer to Chapter 10 of Yunus Cengel and John Cimbala's Fluid Mechanics [1], Chapter 22 on Stokes and Oseen Flows from Theoretical Hydrodynamics 5th Edition by L. M. Milne Thomson [2], and Chapter 2 in Low Reynolds Number Hydrodynamics by J. Happel and H. Brenner [3].

### Note B

A graphical user interface (GUI) for HYDRO++ program, WinHydro++, can be found on the HYDRO++ website (http://leonardo.inf.um.es/macromol/programs/hydro++/hydro++.htm). The v1.0 version of WinHydron++ is used in our paper. Briefly, in order to access the parallel and perpendicular translational drag coefficients, "Full Diffusion Tensors" option should be enabled. The ratio of translational drag coefficients is calculated in the 3x3 Dtt (translational diffusion tensor) box of the 6x6 trans+rot diffusion matrix. More information on the matrix calculation used in Hydro++ is available in [4] and [5].

### Figure A

The image processing algorithm for determining and tracking the position of magnetic particles is written in LabVIEW program mainly using NI Vision Development Module (Fig A).

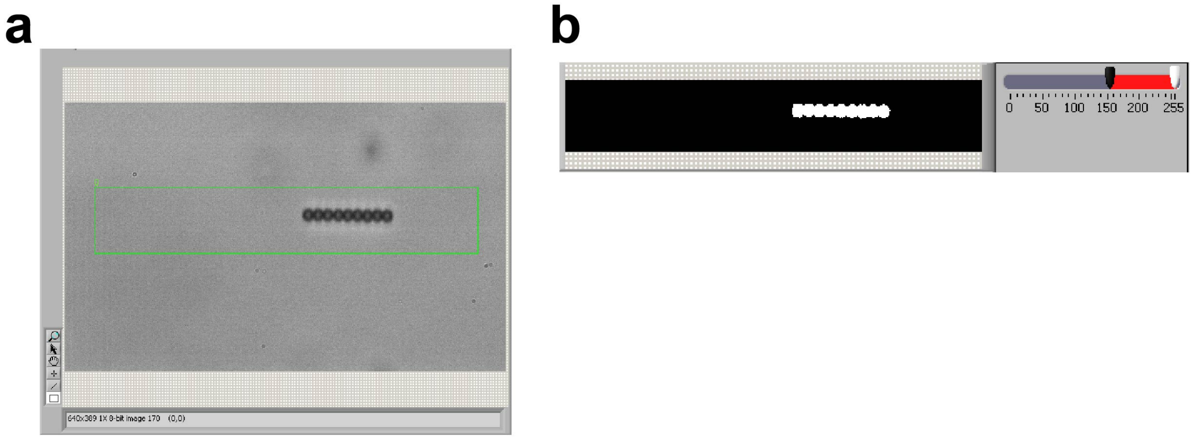


**Fig A. Image processing for tracking of magnetic particles in LabVIEW.** (a): Acquiring the particle images using CCD camera, then selecting the region of interest (ROI) as shown in the green overlay rectangular region in camera view. (b): Pixel information in the ROI is then converted to binary image using threshold for grayscale values. The position of the particle is computed by IMAQ Centroid. For every frame acquired, the centroid position of the binary feature is updated in each iteration.

## S1 Video (screenshot displayed)

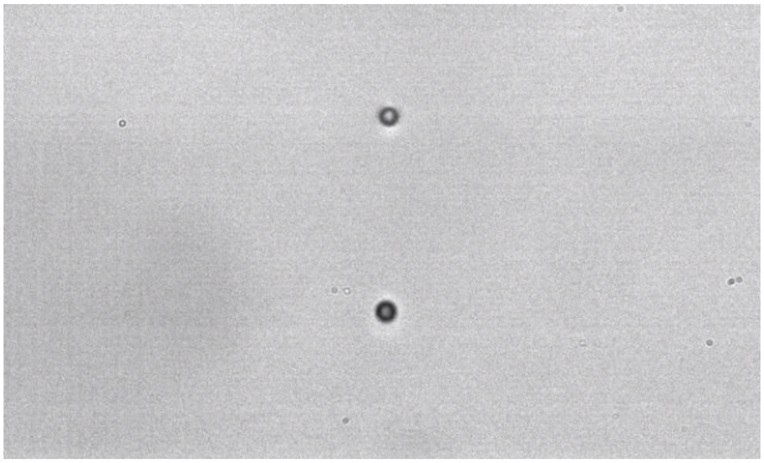

Translational motion of two 2.8 µm-diameter single beads. These two beads happen to be on slightly different focal plane, thus the different darkness. They are pulled towards the magnet which is to the left side of the field of view. Their velocities change according to the distance towards the magnet.

## S2 Video (screenshot displayed)

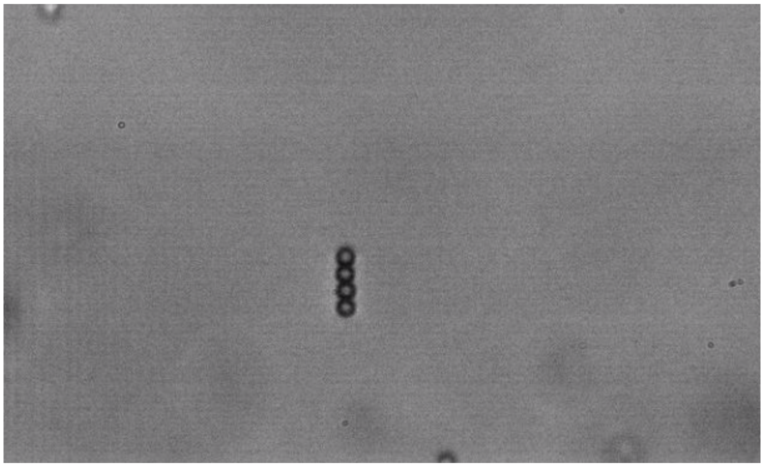

Perpendicular translation of beads and bead-chains. Shown in this video is a magnetic bead-chain of length $N = 4$ translating in the magnetic field by a pair of anti-parallel cylindrical magnets.

## S3 Video (screenshot displayed)

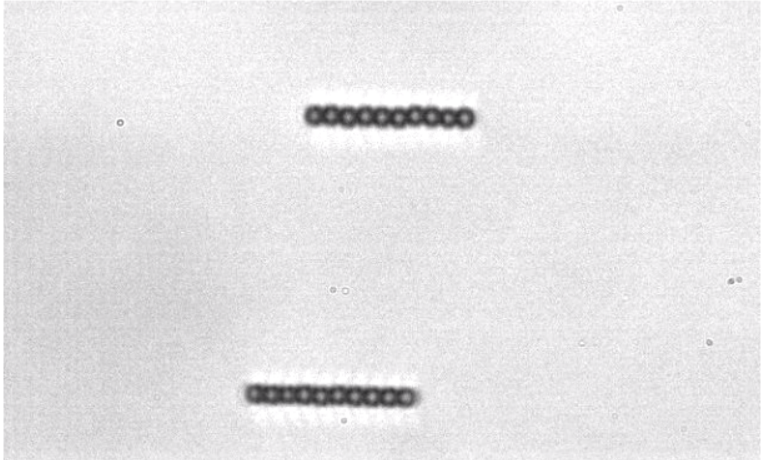

Parallel translation of two bead-chains of length $N = 10$ in the magnetic field due to a cone-shaped magnet.

# References


1. Cengel Y, Cimbala J. Fluid Mechanics Fundamentals and Applications: Third Edition. McGraw-Hill Higher Education; 2013.

2. Milne-Thomson LM. Theoretical hydrodynamics: Fifth Edition. Macmillan Press Ltd.; 1972.

3. Happel J, Brenner H. Low Reynolds number hydrodynamics : with special applications to particulate media. Springer Netherlands; 1981.

4. Carrasco B, de la Torre JG. Hydrodynamic properties of rigid particles: comparison of different modeling and computational procedures. Biophysical journal. 1999;76(6):3044–3057.

5. García de la Torre J, del Rio Echenique G, Ortega A. Improved calculation of rotational diffusion and intrinsic viscosity of bead models for macromolecules and nanoparticles. The Journal of Physical Chemistry B. 2007;111(5):955–961.
